# Imaging diagnosis of adult-type diffuse gliomas in the era of WHO CNS5 and cIMPACT-NOW updates 8–11: a narrative review

**DOI:** 10.1007/s11604-026-02013-6

**Published:** 2026-05-20

**Authors:** Ryo Kurokawa, Hirokazu Takami, Mariko Kurokawa, Yuji Ohizumi, Issei Fukuda, Yosuke Kitagawa, Masashi Nomura, Nobuhito Saito, Osamu Abe

**Affiliations:** 1 Department of Radiology, Graduate School of Medicine, The University of Tokyo, 7-3-1 Hongo, Bunkyo-ku, Tokyo, 113-8655 Japan; 2 Department of Neurosurgery, Graduate School of Medicine, The University of Tokyo, 7-3-1 Hongo, Bunkyo-ku, Tokyo, 113-8655 Japan

**Keywords:** World Health Organization, Glioma, Astrocytoma, Oligodendroglioma, Glioblastoma, cIMPACT-NOW

## Abstract

The 2021 World Health Organization Classification of Tumours of the Central Nervous System, 5th edition (WHO CNS5) reorganized adult-type diffuse gliomas into three integrated tumor types—astrocytoma, IDH-mutant (AST); oligodendroglioma, IDH-mutant and 1p/19q-codeleted (ODG); and glioblastoma, IDH-wildtype (GBM)—and introduced molecular criteria that allow histologically lower-grade IDH-wildtype astrocytic tumors to be diagnosed as GBM (so-called “molecular” GBM). Although molecular genetic information has become central to glioma diagnosis, preoperative imaging-based prediction of tumor type remains essential for guiding surgical strategy, the choice between maximal safe resection and diagnostic biopsy, and treatment planning before final integrated diagnosis. The recent INDIGO trial, which demonstrated efficacy of the IDH1/2 inhibitor vorasidenib in residual grade 2 IDH-mutant glioma, has further increased the importance of preoperative imaging assessment of IDH mutation status. This mini-review summarizes the current role of imaging in adult-type diffuse gliomas in the WHO CNS5 era. First, we review imaging differentiation between AST and ODG, including the T2-fluid-attenuated inversion recovery (FLAIR) mismatch sign and its partial form, classic features supporting ODG such as coarse calcification and cortical-subcortical location, and newer biomarkers including the cortical high-flow sign on arterial spin labeling, subcortical FLAIR signal drop, and ^1^H-MR spectroscopy findings such as 2-hydroxyglutarate and cystathionine. Second, we describe imaging features of “molecular” GBM, including its frequently infiltrative, poorly enhancing appearance and longitudinal histologic grade progression. Third, we discuss the implications of cIMPACT-NOW Updates 8–11 for neuroradiologic diagnosis. In the WHO CNS5 era, imaging plays a central role in supporting integrated diagnosis and prioritizing appropriate molecular testing.

## Introduction

The 2016 revision of the WHO Classification of Tumours of the Central Nervous System formally introduced integrated diagnosis by incorporating molecular genetic alterations, such as IDH mutation and 1p/19q codeletion, into the definition of tumor types [1]. WHO CNS5 further developed this framework by reorganizing adult-type diffuse gliomas into three tumor types: astrocytoma, IDH-mutant (AST); oligodendroglioma, IDH-mutant and 1p/19q-codeleted (ODG); and glioblastoma, IDH-wildtype (GBM). WHO CNS5 also clarified grading within tumor types, the use of the term CNS WHO grade, essential and desirable diagnostic criteria, the separate classification of pediatric-type diffuse gliomas, and the diagnostic role of DNA methylation profiling [2–5]. AST may now be assigned CNS WHO grade 2, 3, or 4, and the former diagnoses of “anaplastic astrocytoma” and “glioblastoma, IDH-mutant” have been abolished. A diagnosis of ODG requires demonstration of both IDH mutation and 1p/19q codeletion. Under WHO CNS5, an IDH-wildtype diffuse astrocytic glioma is diagnosed as GBM, when it shows histologic grade 4 features such as necrosis or microvascular proliferation. Even in the absence of such histologic grade 4 features, GBM can be diagnosed when EGFR amplification, TERT promoter mutation, or combined whole chromosome 7 gain and whole chromosome 10 loss (+ 7/−10) is present. So-called “molecular” GBM refers to tumors that are histologically lower grade, that is, grade 2 or 3, but are classified as GBM on the basis of molecular genetic criteria. Depending on the cohort and criteria used, molecular GBM has been reported to account for more than 60%–70% of histologically lower-grade IDH-wildtype diffuse gliomas [6]. However, recent refinements have been proposed by cIMPACT-NOW Update 11 [7]. Specifically, caution is recommended when assigning CNS WHO grade 4 or a diagnosis of glioblastoma, IDH-wildtype, to a “TERT promoter-only,” histologically low-grade, IDH-wildtype tumor, and EGFR amplification alone or + 7/−10 alone should not be used as solitary defining features for diagnosing glioblastoma, IDH-wildtype, in patients younger than 40 years. These proposed refinements emphasize that the WHO CNS5 molecular criteria for GBM should be interpreted in the context of age, histology, H3 status, and the broader differential diagnosis, particularly pediatric-type diffuse high-grade glioma, H3-wildtype and IDH-wildtype.

As molecular genetic information has become increasingly important, it might appear that the role of preoperative imaging in brain tumor diagnosis has diminished. This impression is misleading. The three types of adult-type diffuse glioma differ substantially in prognosis, surgical strategy, postoperative chemoradiotherapy, and eligibility for molecular targeted therapy. Importantly, the value of preoperative imaging is not simply to avoid diagnostic biopsy. Diagnostic biopsy remains appropriate, and is sometimes preferable, for deep-seated lesions, tumors located in eloquent areas, multifocal or diffuse lesions, or when the differential diagnosis includes primary central nervous system lymphoma, demyelinating disease, infection, metastasis, or other non-glioma entities [8, 9]. Rather, an imaging-based diagnostic hypothesis helps optimize tissue acquisition by identifying the most radiologically aggressive target for biopsy, such as regions with focal enhancement, diffusion restriction, elevated perfusion, hyperattenuation on non-enhanced CT, or metabolic abnormality [10]. Even when maximal safe resection is planned, preoperative imaging can guide spatially annotated tissue sampling, allowing specimens from regions predicted to harbor the highest-grade component to be separately labeled and submitted for neuropathologic and molecular evaluation. Imaging-based prediction also informs the expected extent of resection, the need for awake surgery or functional mapping, and the selection of the most appropriate surgeon and multidisciplinary team. In addition, anticipating the molecular subtype before surgery may support early screening for molecularly stratified clinical trials or targeted therapies and enables more precise preoperative counseling regarding prognosis and treatment strategy [11]. Thus, preoperative imaging-based tumor type prediction should be regarded as a tool for optimizing biopsy targeting, surgical planning, specimen handling, molecular testing, clinical-trial readiness, and patient-centered decision making before the final integrated diagnosis.

The recent demonstration in the international phase 3 INDIGO trial that the IDH1/2 inhibitor vorasidenib significantly prolonged progression-free survival in patients with postoperative grade 2 IDH-mutant glioma with residual disease further increases the importance of preoperative imaging-based assessment of IDH mutation status [12].

This review discusses the current role of imaging in adult-type diffuse gliomas, focusing on imaging differentiation between AST and ODG, imaging diagnosis of so-called “molecular” GBM, and the implications of cIMPACT-NOW Updates 8–11 after WHO CNS5 [7, 13–15].

## Imaging differentiation between astrocytoma, IDH-mutant, and oligodendroglioma, IDH-mutant and 1p/19q-codeleted

**Fig. 1:**
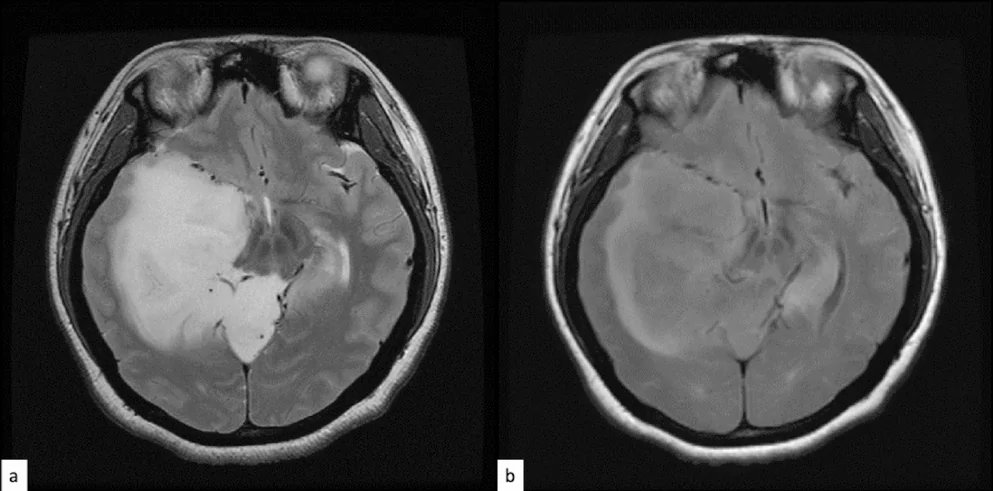
Astrocytoma, IDH-mutant, CNS WHO grade 3 in a 26-year-old woman. The tumor mass in the right temporal lobe shows hyperintensity on T2-weighted image (**a**) and hypointensity on fluid-attenuated inversion recovery (FLAIR) image with the peripheral hyperintense rim (**b**). The T2-FLAIR mismatch sign is positive in this case

**Fig. 2:**
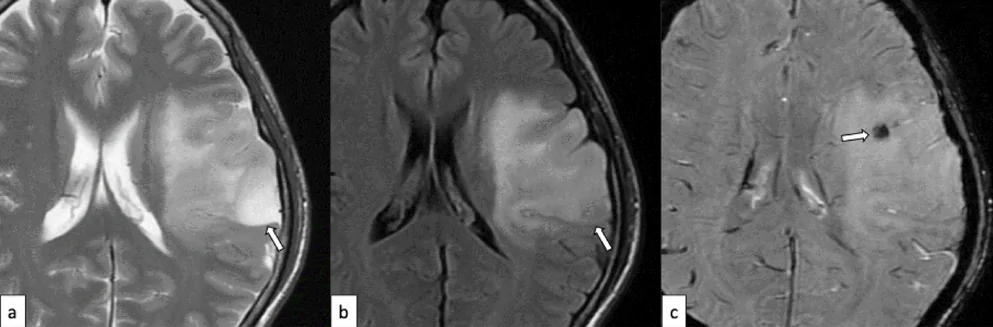
Astrocytoma, IDH-mutant, CNS WHO grade 4 in a 55-year-old woman. The tumor mass shows hyperintensity on T2-weighted image (**a**), both hyperintensity and iso–hypointensity on fluid-attenuated inversion recovery (FLAIR) image (**b**), with evidence of intratumoral hemorrhage on susceptibility-weighted image (**c**, arrow). Partial T2-FLAIR mismatch sign is positive in this case (**a**,** b**, arrows)

The T2-fluid-attenuated inversion recovery (FLAIR) mismatch sign refers to a lesion that is complete or nearly complete hyperintense on T2-weighted imaging and complete or nearly complete signal drop on FLAIR, except for a hyperintense peripheral rim (Fig. 1). It is now established as a highly specific imaging biomarker for IDH-mutant, 1p/19q-non-codeleted astrocytoma among adult-type diffuse gliomas [16]. Its specificity was first demonstrated using TCGA/TCIA data, and a subsequent systematic review and meta-analysis supported its diagnostic profile as a sign with very high specificity but limited sensitivity (pooled sensitivity, 0.42; pooled specificity, 0.99) [17]. Histopathologic-radiologic correlation studies suggest that the T2-FLAIR mismatch sign reflects region-dependent microstructural differences, including microcystic change and myxoid matrix [18]. Partial T2-FLAIR mismatch, in which only part of the tumor shows the mismatch pattern, has also attracted attention (Fig. 2). In a study of WHO CNS grade 4 diffuse astrocytic tumors in adults, partial T2-FLAIR mismatch was significantly more frequent in the IDH-mutant group, corresponding to astrocytoma, IDH-mutant, CNS WHO grade 4, than in the IDH-wildtype group, corresponding to GBM (32/121 [26.4%] vs. 8/2044 [0.4%]) [19]. These findings suggest that a strictly all-or-none classical definition may fail to identify a subset of AST on preoperative imaging.

**Fig. 3:**
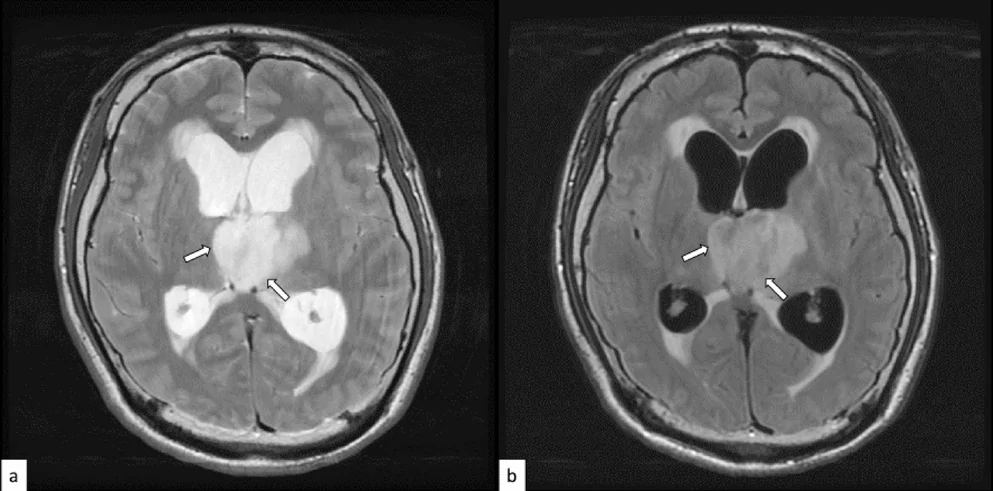
Diffuse midline glioma, H3 K27-altered in a 36-year-old man. The thalamic tumor mass shows hyperintensity on T2-weighted image (**a**) and hypointensity on fluid-attenuated inversion recovery (FLAIR) image (**b**). The T2-FLAIR mismatch is observed in this case

Two caveats are essential when interpreting the T2-FLAIR mismatch sign. First, signal changes corresponding to cystic degeneration or necrosis should not be judged as positive. For example, interpreting the necrotic component of GBM as T2-FLAIR mismatch may lead to a false-positive diagnosis in IDH-wildtype disease. Second, the T2-FLAIR mismatch sign is a highly specific imaging clue for AST, or for IDH-mutant status, only within the appropriate clinical and imaging context of adult-type diffuse glioma; it is not a stand-alone diagnostic criterion for tumor type. A shortcut diagnosis of AST simply because a mismatch-like appearance is present is therefore unsafe. T2-FLAIR mismatch can be seen in other tumor categories, including pediatric-type gliomas such as diffuse midline glioma, H3 K27-altered (Fig. 3); diffuse astrocytoma, MYB- or MYBL1-altered; angiocentric glioma; diffuse low-grade glioma, MAPK pathway-altered; pilocytic astrocytoma; and dysembryoplastic neuroepithelial tumor, as well as in non-neoplastic disorders such as demyelinating disease [5, 20, 21].

**Fig. 4:**
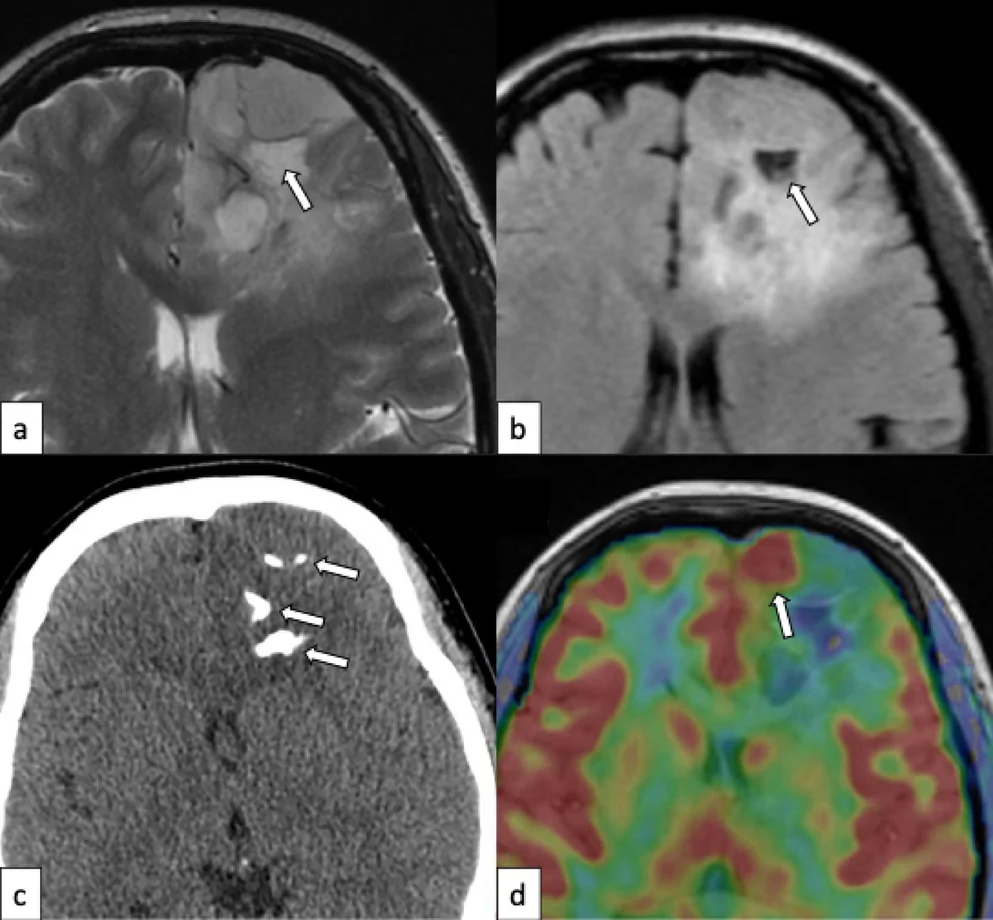
Oligodendroglioma, IDH-mutant and 1p/19q-codeleted, CNS WHO grade 3 in a 43-year-old woman. The tumor mass located in the cortico-subcortical region in the left frontal lobe shows hyperintensity on T2-weighted image (**a**) and on fluid-attenuated inversion recovery (FLAIR) image (**b**). Subcortical FLAIR signal drop is positive in this case (**a**,** b**, arrows). Coarse calcification is observed within the tumor mass on non-enhanced computed tomography (**c**, arrows). Cortical high-flow sign is observed on arterial spin labeling (**d** (fusion image), arrow)

Classic imaging features that support ODG rather than AST include coarse calcification, cortical-subcortical location, and frontal lobe predominance; these remain useful differentiating features (Fig. 4) [22]. More recently, the cortical high-flow sign on arterial spin labeling (ASL) has been reported as a supportive imaging feature of ODG [23, 24]. In a 71-case study by Yamashita et al. [23], this sign was present in 12/22 ODGs (54.5%), 2/28 ASTs (7.1%), and 2/21 IDH-wildtype gliomas (9.5%). For differentiating ODG from AST, these figures correspond to a sensitivity of 54.5% and a specificity of 92.9%.

Subcortical FLAIR signal drop, defined as FLAIR signal decrease localized to subcortical white matter, has also been reported as a new imaging feature supporting ODG. In a study by Ohizumi et al. [25] of 41 IDH-mutant adult-type diffuse gliomas (16 ASTs and 25 ODGs), subcortical FLAIR signal drop was observed in 1/16 ASTs (6.3%) and 11/25 ODGs (44.0%), yielding a sensitivity of 44.0% and a specificity of 93.8% for predicting ODG. Furthermore, the presence of at least two ODG-favored features among subcortical FLAIR signal drop, multiple cystic/necrotic changes, and calcification achieved 100% specificity for ODG, whereas the presence of T2-FLAIR mismatch without ODG-favored features achieved 96.0% specificity for AST [25].

Proton MR spectroscopy (1 H-MRS) may also assist in preoperative molecular subtype prediction in adult-type diffuse gliomas. 2-Hydroxyglutarate (2HG) is an oncometabolite that accumulates as a consequence of IDH1/2 mutation, and detection of 2HG by MRS is useful for noninvasive inference of IDH mutation [26]. In contrast, cystathionine tends to accumulate in 1p/19q-codeleted gliomas and has been reported as a metabolic marker supporting ODG [27]. In an 85-case study of adult-type diffuse gliomas by Kikuchi et al. [28] (25 ODGs, 28 ASTs, and 32 GBMs), cystathionine concentration was highest in ODG and significantly higher in ODG than in AST. However, because of overlap with GBM, the diagnostic performance of cystathionine alone was limited, with an area under the curve of 0.69 for ODG versus AST and 0.56 for ODG versus GBM [28]. In another study of IDH-mutant adult-type diffuse gliomas, combining the cortical high-flow sign with cystathionine increased the area under the curve for differentiating ODG from AST to 0.875. Thus, MRS should be regarded not as a stand-alone determinant but as metabolic information that complements MRI, CT, and ASL findings [29].

## Molecular glioblastoma, IDH-wildtype

“Molecular” GBM is not an official WHO tumor name. Rather, it is an informal term referring to tumors that, under WHO CNS5 criteria, lack histologic features of grade 4 disease such as necrosis and microvascular proliferation and therefore show a histologically lower-grade appearance, but are diagnosed as GBM because they harbor molecular criteria such as EGFR amplification, TERT promoter mutation, or + 7/−10. Because these tumors may show little necrosis or contrast enhancement on CT or MRI, a major pitfall is to underestimate tumor aggressiveness and mistake them for lower-grade tumors. Nevertheless, their prognosis is generally poor, similar to GBM, although it is heterogeneous depending on histologic grade and the specific molecular criteria present. In a cohort of 1211 adult-type diffuse gliomas, histologically grade 4 GBM accounted for 777 cases (64.2%), histologically grade 2 or 3 GBM, that is, molecular GBM, for 40 cases (3.3%), AST for 204 cases (16.8%), and ODG for 190 cases (15.7%) [30]. Thus, approximately 1 in 20 GBMs in that cohort was a molecular GBM.

**Fig. 5:**
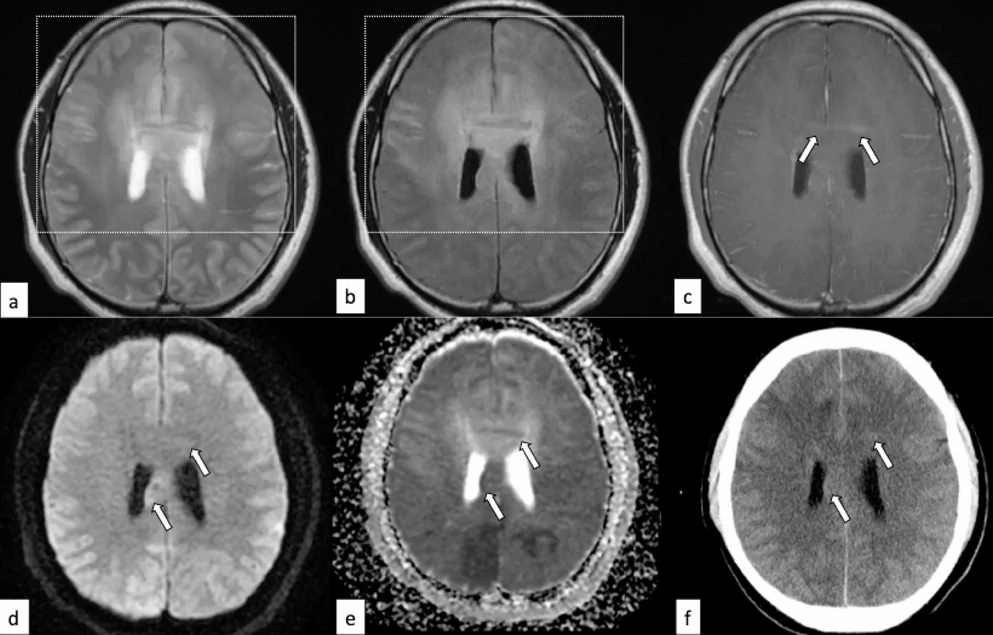
“Molecular” glioblastoma, IDH-wildtype with gliomatosis cerebri growth pattern in a 40-year-old man. There is an infiltrating tumor involving the bilateral frontal and parietal lobes (**a**,** b**, dashed line). The tumor shows hyperintensity on T2-weighted image (**a**) and fluid-attenuated inversion recovery (FLAIR) image (**b**). The majority of the tumor shows no enhancement, although focal areas of enhancement are observed on post-contrast T1-weighted image (**c**, arrows). Focal areas of diffusion restriction (**d** (diffusion-weighted image),** e** (apparent diffusion coefficient map), arrows) and hyperintensity on non-enhanced computed tomography (**f**, arrows) are also observed. Histologically, the tumor lacked both necrosis and microvascular proliferation, corresponding to histological grade 3; however, the presence of a TERT promoter mutation led to the final diagnosis of glioblastoma, IDH-wildtype

The imaging features of molecular GBM can be summarized as follows. The tumor often appears infiltrative and poorly marginated, with absent or faint enhancement. Histologically lower-grade components show predominantly hypoattenuating on non-enhanced CT, show no diffusion restriction, and demonstrate low relative cerebral blood volume. However, focal regions suggesting histologically high-grade components, such as hyperattenuation on non-enhanced CT, diffusion restriction, or increased relative cerebral blood volume, may coexist within the tumor (Fig. 5) [31, 32]. Recognition of such focal aggressive components is clinically important because they may guide targeted biopsy, spatially annotated tissue sampling, and prioritization of molecular testing. Another important imaging feature of molecular GBM is the lack of imaging signs that support classic GBM, AST, or ODG. Furthermore, molecular GBM has been reported to show a gliomatosis cerebri growth pattern in 30% of cases and multifocal distribution in 9% [30].

**Fig. 6:**
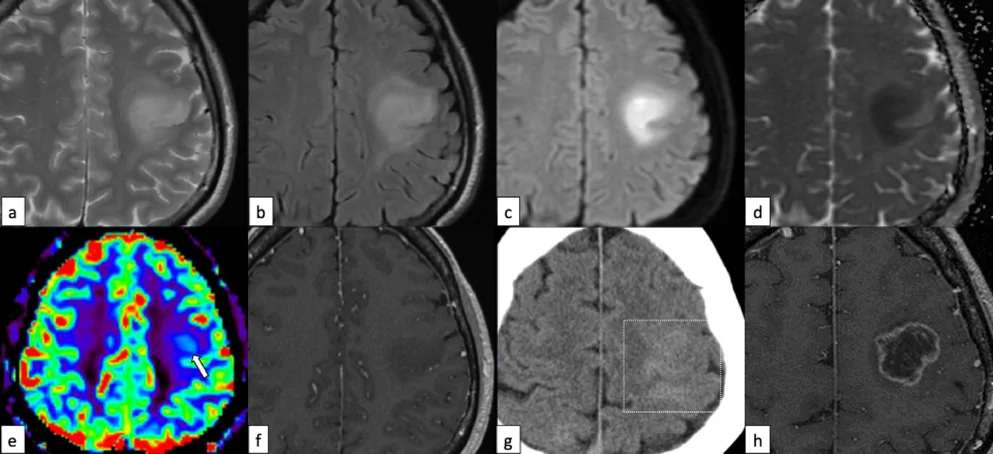
Glioblastoma, IDH-wildtype in a 53-year-old woman. The tumor mass located in the left frontal and parietal lobes shows hyperintensity on T2-weighted image (**a**), fluid-attenuated inversion recovery (FLAIR) image (**b**), and diffusion-weighted image (**c**) with low apparent diffusion coefficient (**d**). Relative cerebral blood volume is low (**e**). Contrast enhancement is not observed within the tumor mass on post-contrast T1-weighted image (**f**). Differential diagnoses included malignant glioma, lymphoma, and demyelinating disease. The lesion was hyperdense on non-enhanced CT (**g**, dashed line), raising suspicion for a malignant brain tumor. One month later, contrast-enhanced MRI revealed ring-like enhancement, which was characteristic of glioblastoma, IDH-wildtype (**h**). These findings were thought to reflect the temporal progression of a molecular glioblastoma from a histologically lower-grade tumor to one meeting histological criteria for grade 4

A frequently overlooked longitudinal imaging feature of molecular GBM is that histologic grade may increase over time or at recurrence [33] (Fig. 6). An important caveat is that, particularly in biopsy-diagnosed cases, histologic grade 4 components such as necrosis or microvascular proliferation may be absent from the biopsy specimen, leading to misclassification as histologically lower-grade disease [33]. Accordingly, molecular GBM should not be diagnosed by accepting biopsy histology in isolation; rather, it should be an integrated diagnosis made after confirming that CT and MRI findings are compatible with the diagnosis.

Gliomatosis cerebri, which is relatively frequent in molecular GBM, was previously regarded as an independent tumor entity. Since the 2016 WHO classification, however, it has been treated not as a separate entity but as a growth pattern of diffuse glioma involving three or more cerebral lobes. Muench et al. [34] recently reported, using DNA methylation profiling, a distinct group termed high-grade glioma subtype F (HGG-F). All 16 HGG-F tumors were IDH-wildtype diffuse gliomas, and none showed histologic necrosis or microvascular proliferation. Contrast enhancement was observed in only 3/16 cases, and none showed the ring-like enhancement typical of GBM [34]. TERT promoter mutation was present in 12/15 cases, meaning that most would meet WHO CNS5 criteria for molecular GBM, whereas EGFR amplification was absent in all cases and + 7/−10 was observed in only 2/16 cases. The median overall survival was 74 months, which is longer than that of typical GBM [34]. HGG-F suggests that some tumors with a gliomatosis cerebri growth pattern may be reclassified not merely as a growth pattern but as an independent DNA methylation class. To provide a practical overview of the subtype-oriented imaging clues discussed above, Table 1 summarizes representative imaging findings and reported diagnostic performance for AST, ODG, and molecular GBM. Because the evidence base differs across studies, sensitivity and specificity are shown when available; otherwise, reported frequencies or area under the receiver operating characteristic curve values are provided.

**Table 1:** Major imaging findings supporting molecular subtype prediction in adult-type diffuse gliomas

Molecular subtype suggested	Representative imaging finding/biomarker	Modality/Sequence	Reported diagnostic performance	Key interpretation	References
AST	Classic T2-FLAIR mismatch sign	T2WI, FLAIR	Pooled sensitivity 0.42; pooled specificity 0.99 for IDH-mutant, 1p/19q-non-codeleted glioma	Highly specific rule-in sign for AST in the appropriate adult-type diffuse glioma context; cystic or necrotic components should not be counted as positive	[16, 17]
AST	Partial T2-FLAIR mismatch sign in CNS WHO grade 4 astrocytic tumors	T2WI, FLAIR	Sensitivity 26.4%; specificity 99.6% for IDH-mutant status among CNS WHO grade 4 astrocytic tumors, calculated from 32/121 IDH-mutant vs. 8/2044 IDH-wildtype cases	Useful high-specificity clue for IDH-mutant astrocytoma even when the mismatch is not near-complete	[19]
AST	Redefined T2-FLAIR mismatch sign, including partial mismatch but excluding subcortical-only mismatch	T2WI, FLAIR	Sensitivity 87.5%; specificity 80.0% for AST vs. ODG	Improves sensitivity for AST compared with strict classic T2-FLAIR mismatch; evidence is from a single-center IDH-mutant adult-type diffuse glioma cohort	[25]
ODG	Coarse calcification, cortical-subcortical location, frontal lobe predominance	MRI, CT	Robust pooled sensitivity/specificity not established; calcification was observed in 50.0% of ODG and 0% of AST in one IDH-mutant adult-type diffuse glioma cohort	Classic supportive features for ODG; CT remains important for detecting calcification	[22, 25]
ODG	Cortical high-flow sign	ASL	Sensitivity 54.5%; specificity 92.9% for ODG vs. AST, calculated from 12/22 ODG and 2/28 AST cases	High-specificity supportive sign for ODG; histologic correlation with cortical vascular density has been reported	[23, 24]
ODG	Subcortical FLAIR signal drop	T2WI, FLAIR	Sensitivity 44.0%; specificity 93.8% for ODG vs. AST	Specific supportive sign for ODG; may mimic partial T2-FLAIR mismatch when mild, so subcortical localization should be carefully assessed	[25]
ODG	Two or more ODG-favored features: subcortical FLAIR signal drop, multiple cystic/necrotic changes, and calcification	T2WI, FLAIR, CT	Sensitivity 48.0%; specificity 100% for ODG vs. AST	Highly specific rule-in combination for ODG in IDH-mutant adult-type diffuse glioma	[25]
ODG	Cystathionine on proton MR spectroscopy, alone or combined with cortical high-flow sign	Proton MR spectroscopy / ASL	Cystathionine alone: AUC 0.69 for ODG vs. AST and 0.56 for ODG vs. GBM; cortical high-flow sign plus cystathionine: AUC 0.875 for ODG vs. AST	Metabolic adjunct rather than stand-alone determinant; useful when integrated with MRI, CT, and ASL findings	[27–29]
Molecular GBM	Poorly marginated infiltrative lesion with absent or faint enhancement; lack of AST- or ODG-supporting features; focal hyperattenuation, diffusion restriction, or elevated rCBV may coexist	MRI, CT	Validated sensitivity/specificity not established	May appear lower-grade on imaging and histology; focal high-grade imaging components should prompt targeted sampling and molecular work-up	[30–33]
Molecular GBM	Gliomatosis cerebri growth pattern	T2WI, FLAIR	Reported in 30% of molecular GBM; count-based sensitivity 30.0% and specificity 91.6% for molecular GBM vs. histologically grade 4 GBM, calculated from 12/40 molecular GBM vs. 65/777 histologically grade 4 GBM cases	Not specific enough as a stand-alone diagnostic sign; useful as a warning pattern when classic AST/ODG features are absent	[30]

AST = astrocytoma, IDH-mutant; ODG = oligodendroglioma, IDH-mutant and 1p/19q-codeleted; GBM = glioblastoma, IDH-wildtype; ASL = arterial spin labeling; FLAIR = fluid-attenuated inversion recovery; rCBV = relative cerebral blood volume; AUC = area under the receiver operating characteristic curve. Values shown as sensitivity and specificity were taken from the original reports when explicitly provided, or calculated from reported counts when appropriate

## cIMPACT-NOW updates 8–11 after WHO CNS5

WHO CNS5 substantially incorporated the recommendations of cIMPACT-NOW Updates 1–7. cIMPACT-NOW, the Consortium to Inform Molecular and Practical Approaches to CNS Tumor Taxonomy—Not Official WHO, consists of international experts in neuropathology, neuroradiology, molecular genetics, clinical oncology, and related fields [35–41]. CNS tumor classification did not, however, end with WHO CNS5; it continues to evolve.

cIMPACT-NOW Update 8 clarified the practical application of meningioma grading. It proposed a standardized interpretation of brain invasion and recommended grading reassessment that incorporates not only histology but also molecular risk parameters, including 1p deletion together with 22q deletion or NF2 alteration, TERT promoter mutation, and CDKN2A/B homozygous deletion [13].

Update 9 clarified the diagnostic role of DNA methylation profiling. It emphasized that methylation profiling results should not be interpreted as self-contained outputs but should be integrated into a layered diagnosis in the context of imaging, clinical information, histology, and other molecular genetic findings [14]. It also emphasized that interpretation should include not only methylation class but also copy-number profile and auxiliary biomarkers.

Update 10 proposed criteria for introducing new tumor types into future WHO classifications. It indicated that a distinct clinical phenotype, molecular clustering, driver alterations, microscopic and immunohistochemical features, and characteristic imaging findings may all contribute to the definition of a new tumor type [15]. Thus, imaging is not merely ancillary to taxonomy; rather, characteristic imaging findings may be integral to the establishment of tumor types.

Update 11 proposed revisions to the diagnostic criteria for glioblastoma, IDH-wildtype, by clarifying that the relevant diagnostic category is an IDH-wildtype and H3-wildtype diffuse astrocytic glioma [7]. It proposed that isolated TERT promoter mutation alone should not be sufficient to diagnose a histologically low-grade tumor as GBM. This recommendation refocuses attention on the long-standing concern that molecular GBMs defined only by TERT promoter mutation, including many tumors resembling the HGG-F group described above, may have a significantly better prognosis than other GBMs and should not necessarily be grouped with them without nuance [42]. Update 11 also proposed that EGFR amplification alone or + 7/−10 alone should not be used to diagnose GBM in patients younger than 40 years, because these alterations may also be seen in non-adult-type tumor groups that are more frequent in younger patients, including pediatric-type diffuse gliomas, reducing specificity.

Update 11 further stated that, in non-midline tumors in patients older than 55 years, H3 K27 testing may be omitted; and retained ATRX expression together with OLIG2 positivity supports H3 G34-wildtype status [7]. These points are important for practical diagnostic algorithms that distinguish GBM from diffuse pediatric-type high-grade glioma, H3-wildtype and IDH-wildtype. Update 11 also emphasized that, for diffuse pediatric-type high-grade glioma, H3-wildtype and IDH-wildtype, which can be difficult to distinguish from GBM, definitive diagnosis requires DNA methylation profiling. Especially in young adults with IDH-wildtype/H3-wildtype diffuse high-grade glioma, pediatric-type HGG methylation classes should be considered before simply classifying the tumor as adult-type GBM. PDGFRA alteration, EGFR alteration, and MYCN amplification may support diffuse pediatric-type high-grade glioma, H3-wildtype and IDH-wildtype, but Update 11 mainly restricts the use of these as diagnostic features to patients younger than 25 years [7].

Update 11 also proposed diagnostic refinements for posterior fossa ependymal tumors, including PFA/PFB ependymoma and related methylation classes [7]. Although these tumor types are beyond the main focus of this review, they provide another important example of how post-WHO CNS5 tumor classification continues to evolve around DNA methylation profiling.

## Conclusion

In the era of WHO CNS5 and cIMPACT-NOW Updates 8–11, the value of imaging has not declined. Rather, imaging has become even more important as an indispensable component of integrated diagnosis. The T2-FLAIR mismatch sign, particularly redefined or partial T2-FLAIR mismatch not confined to subcortical white matter, is an important rule-in sign for AST. Cortical-subcortical location, frontal predominance, coarse calcification, the cortical high-flow sign, subcortical FLAIR signal drop, and multiple cystic or necrotic changes support ODG. Conversely, when a poorly marginated infiltrative tumor with absent or faint enhancement lacks imaging features that actively support AST or ODG, molecular GBM should be considered even if the lesion appears similar to a lower-grade glioma. Raising suspicion for specific molecular subtypes and prioritizing appropriate molecular tests based on imaging findings are now central roles of neuroradiologic diagnosis in brain tumor care.

## Data Availability

Original data was not generated for this study.

## Abbreviations

AST: Astrocytoma, IDH-mutant
ASL: Arterial spin labeling
AUC: Area under the receiver operating characteristic curve
FLAIR: Fluid-attenuated inversion recovery
GBM: Glioblastoma, IDH-wildtype
ODG: Oligodendroglioma, IDH-mutant and 1p/19q-codeleted
WHO CNS5: The 2021 World Health Organization Classification of tumours of the central nervous system, 5th edition
